# Platelet-Rich Therapies in Hernia Repair: A Comprehensive Review of the Impact of Platelet Concentrates on Mesh Integration in Hernia Management

**DOI:** 10.3390/biom14080921

**Published:** 2024-07-29

**Authors:** Elissavet Anestiadou, Efstathios Kotidis, Ioanna Abba Deka, Dimitrios Tatsis, Chryssa Bekiari, Antonia Loukousia, Orestis Ioannidis, Stavros Stamiris, Konstantinos Zapsalis, Christos Xylas, Konstantinos Siozos, Christiana Chatzianestiadou, Stamatios Angelopoulos, Theodosios Papavramidis, Angeliki Cheva

**Affiliations:** 1 4th Department of Surgery, General Hospital “George Papanikolaou”, Aristotle University of Thessaloniki, 57010 Exochi, Greece; skotidis@auth.gr (E.K.); telonakos@hotmail.com (O.I.); kzapsal@auth.gr (K.Z.); cxylas@auth.gr (C.X.); ksiozos@auth.gr (K.S.); cchristia@auth.gr (C.C.); saggelopoulos@auth.gr (S.A.); 2 Pathology Department, Faculty of Medicine, Aristotle University of Thessaloniki, 54124 Thessaloniki, Greece; ampanteka@auth.gr (I.A.D.); tonialoukousia@gmail.com (A.L.); antacheva@auth.gr (A.C.); 3 Department of Oral and Maxillofacial Surgery, General Hospital “George Papanikolaou”, Aristotle University of Thessaloniki, 57010 Exochi, Greece; dimitats@auth.gr; 4 Experimental and Research Center, Papageorgiou General Hospital of Thessaloniki, 56403 Thessaloniki, Greece; chmpekia@vet.auth.gr; 5 Laboratory of Anatomy and Histology, Veterinary School, Aristotle University of Thessaloniki, 54124 Thessaloniki, Greece; 6 Orthopaedic Department, 424 General Military Hospital, Ring Road West, Nea Efkarpia, 56429 Thessaloniki, Greece; sstamiris@auth.gr; 7 1st Propaedeutic Department of Surgery, Medical School, Aristotle University of Thessaloniki, 54124 Thessaloniki, Greece; tpapavra@auth.gr

**Keywords:** platelet-rich plasma, platelet-rich fibrin, tissue regeneration, wound healing, incorporation, hernia, abdominal wall, hiatal hernia, mesh, scaffold, synergistic effect, biomaterials

## Abstract

Mesh-augmented hernia repair is the gold standard in abdominal wall and hiatal/diaphragmatic hernia management and ranks among the most common procedures performed by general surgeons. However, it is associated with a series of drawbacks, including recurrence, mesh infection, and adhesion formation. To address these weaknesses, numerous biomaterials have been investigated for mesh coating. Platelet-rich plasma (PRP) is an autologous agent that promotes tissue healing through numerous cytokines and growth factors. In addition, many reports highlight its contribution to better integration of different types of coated meshes, compared to conventional uncoated meshes. The use of PRP-coated meshes for hernia repair has been reported in the literature, but a review of technical aspects and outcomes is missing. The aim of this comprehensive review is to report the experimental studies investigating the synergistic use of PRP and mesh implants in hernia animal models. A comprehensive literature search was conducted across PubMed/Medline, Web of Science, and Scopus without chronological constraints. In total, fourteen experimental and three clinical studies have been included. Among experimental trials, synthetic, biologic, and composite meshes were used in four, nine, and one study, respectively. In synthetic meshes, PRP-coating leads to increased antioxidant levels and collaged deposition, reduced oxidative stress, and improved inflammatory response, while studies on biological meshes revealed increased neovascularization and tissue integration, reduced inflammation, adhesion severity, and mechanical failure rates. Finally, PRP-coating of composite meshes results in reduced adhesions and improved mechanical strength. Despite the abundance of preclinical data, there is a scarcity of clinical studies, mainly due to the absence of an established protocol regarding PRP preparation and application. To this point in time, PRP has been used as a coating agent for the repair of abdominal and diaphragmatic hernias, as well as for mesh fixation. Clinical application of conclusions drawn from experimental studies may lead to improved results in hernia repair.

## 1. Introduction

Abdominal wall hernias are a common clinical entity, presenting a prevalence of 1.7% for all ages pooled and 4% for patients older than 45 years. The majority of abdominal wall hernias, approximately 75%, are hernias of the inguinal region, including inguinal and femoral hernias, while umbilical, paraumbilical, epigastric, incisional, Spigelian, and post-trauma hernias are also frequent in clinical practice. Surgical repair is the management option of choice, especially in cases of evident symptomatology [1]. More than 611,000 ventral and 1 million inguinal hernia repairs are performed annually in the USA, according to a retrospective cohort study based on National Inpatient Sample (NIS) and the Nationwide Ambulatory Surgery Sample (NASS) data [2]. In addition, an increasing trend of emergent hernia repairs has been noticed, from 16.0 to 19.2 per 100,000 person-years from 2001 to 2010, and significant predominance among patients 65 years and older [3].

**Figure 1:**
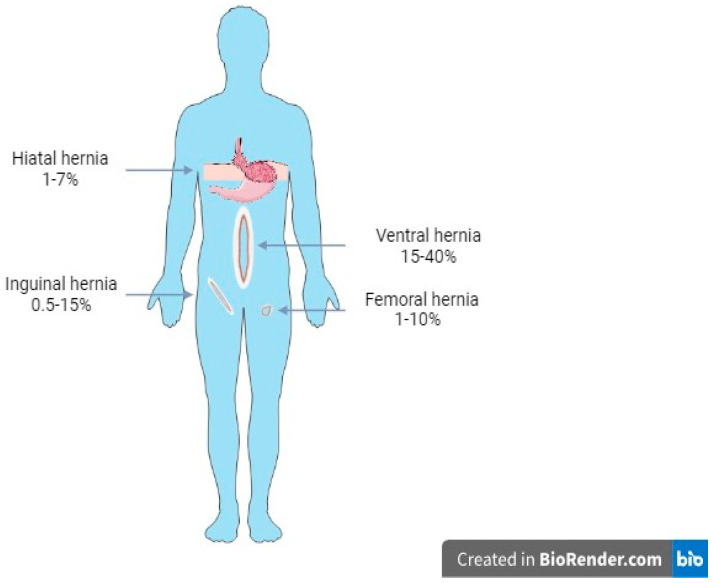
Rates of hernia recurrence according to anatomic site.

Hernia recurrence also constitutes an increasing burden affecting abdominal core health, with an adjusted cumulative incidence of reoperation of approximately 16.1% at 10 years postoperatively [4]. According to the initial anatomic location, the actual rates of hernia recurrence are approximately 0.5% to 15% for inguinal hernias, 1% to 10% for femoral hernias, 15% to 40% for ventral hernias, and 1% to 7% for hiatal hernias, depending upon factors such use of mesh or primary repair, size of the defect, open, laparoscopic or robotic repair, surgeon’s experience and clinical setting, elective or emergent (Figure 1) [5,6,7,8]. Notably, the failure rate of re-recurrent inguinal hernias, defined as a recurrence of a hernia with at least two previous surgical repair procedures, remains alarmingly high, reaching an incidence of 36% [9]. The development of hernia recurrence has been associated with numerous predisposing risk factors, including patient-related factors, surgical risk factors, and/or implant-related factors, in cases of mesh-augmented hernia repair [10]. Among patient-related risk factors for hernia recurrence, factors such as female sex, direct inguinal, incisional, and parastomal hernias, repair of a recurrent inguinal hernia, smoking, size of hernia, obesity, diabetes mellitus, white race, chronic pulmonary disease, multidrug therapy, peripheral vascular disease, and immunosuppression, are included [10,11,12]. Regarding risk factors associated with surgical technique, lower midline incision, bilateral repair, wound infection, wrong mesh size, repair under local anesthesia, limited surgeon and center experience, and use of biologic or resorbable synthetic meshes are surgical risk factors that should be taken into consideration to minimize rates of recurrence after hernia repair [13,14]. Finally, mechanical strain, poor tissue–mesh integration, and degradation of matrices are included in implant-related factors contributing to surgical failure and hernia recurrence [6].

Industry has also played a major role in advancing implant mesh technology, aiming to establish the optimal repair technique and used materials [15]. The application percentage of prosthetic meshes in hernia repair has more than doubled compared to the 1980s. However, the emergence of prosthetic materials has led to only a 24–50% reduction in recurrence rate compared to mesh-free repair [16]. No consensus has been reached regarding the ideal mesh type, as well as the potentially beneficial role of growth factors and cell therapies in improving mesh properties and safety profile [17].

Growth factors promote tissue repair via a series of mechanisms, including cell chemotaxis and proliferation and stimulation of extracellular matrix deposition [18]. Platelet-rich plasma (PRP), defined as an autologous concentration of platelets that is 3 to 5 times higher than the normal concentration in whole blood, is characterized by a high percentage of growth factors, secretory proteins, and other biomolecules necessary for all stages of wound healing [19].

The present comprehensive review aims to report the experimental studies that investigate the synergistic use of PRP products and mesh implants in animal models of hernia repair. The technical aspects of experimental procedures, as well as the results and limitations of each study, are also reported. In addition, reference is made to the limited clinical studies investigating the combined use of PRP and meshes in humans for hernia repair.

### 1.1. Platelet-Rich Plasma Products

**Figure 2:**
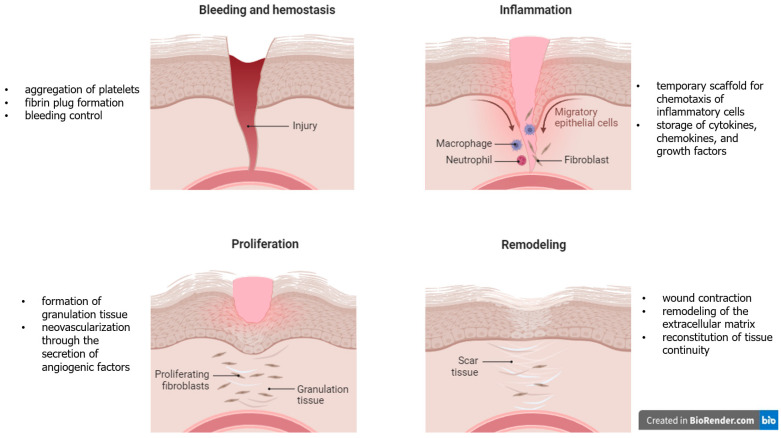
Role of platelets in different phases of wound healing.

Platelets, also called thrombocytes, derive from the bone marrow and have a crucial role in primary hemostasis and thrombosis [20]. However, a series of additional functions of platelets have also been reported, including innate immunity and adaptive immunity responses, inflammation, angiogenesis, stem cell migration, cancer metastasis, and cell proliferation [21]. Apart from the functions above, platelets contribute significantly to all phases of wound healing (Figure 2). More specifically, during hemostasis, aggregation of platelets leads to fibrin plug formation and bleeding control. During the inflammatory phase, platelets provide a temporary scaffold for the chemotaxis of inflammatory cells and storage of cytokines, chemokines, and growth factors. The proliferative phase follows, including mainly the formation of granulation tissue, as well as neovascularization through the secretion of angiogenic factors by platelets. Finally, during the remodeling/maturation phase, platelets contribute to wound contraction, remodeling of the extracellular matrix, and reconstitution of tissue continuity [22]. The increasing knowledge regarding the role of platelets in wound healing resulted in emerging research on the role of platelet-rich plasma (PRP) products in tissue healing and regeneration [23].

**Figure 3:**
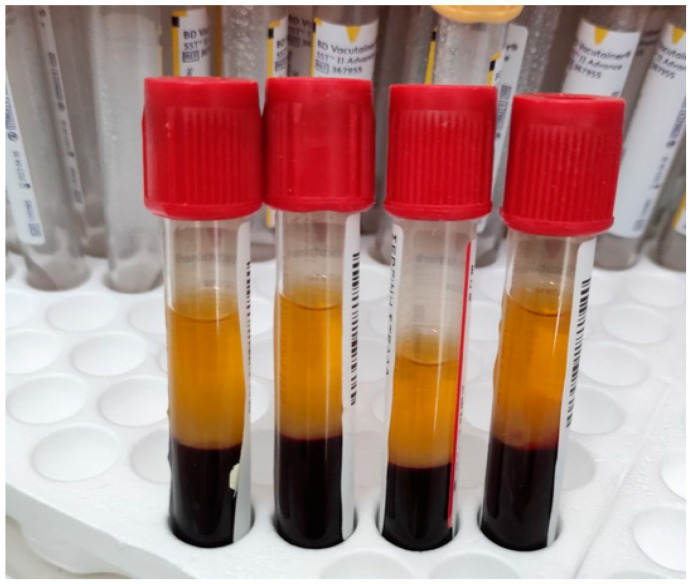
The final product of centrifugation, with PRP being the bottom layer.

Platelet-rich plasma (PRP), which is also known under the terms platelet-rich growth factors (GFs), platelet-rich fibrin (PRF) matrix, PRF, and platelet concentrate, was first introduced in clinical practice in the 1970s to describe a transfusion product for the management of thrombocytopenia [24]. Wide application of PRP in surgical procedures, such as maxillofacial and plastic surgery, began in the 1980s and 1990s [25]. Platelets contain at least three types of granules: dense granules, α-granules, and lysosomes, while presence of peroxisomes and recently described T granules [26]. PPR is an autologous product derived from plasma rich in platelets obtained from whole venous blood. The literature contains a vast variation of published preparation protocols, thus leading to heterogeneity regarding clinical results [27]. The upper layer produced after the first centrifugation represents the rich in platelets plasma, which can be used directly or can undergo a second centrifugation with a higher speed to obtain pure PRP [27]. The final product is activated with the use of calcium chloride (CaCl_2_), autologous thrombin, a mixture of CaCl_2_ and thrombin or collagen type I, in order to trigger degranulation of platelets and secretion of growth factors from α-granules, which is the main mechanism of PRP action (Figure 3) [27]. Similarly to the in vivo wound healing process after contact with exposed collagen, activation of PRP leads to the release of large amounts of a series of growth factors and platelet cytokines with angiogenic, regenerative, and chemotactic roles from α-granules, the most important of them being reported in Table 1. These growth factors enhance the early proliferative phase of wound healing by promoting stem cell proliferation and angiogenesis [27]. The absence of numerous proteins excreted by platelets has been found in chronic wounds, thus underlying their crucial role in tissue healing and regeneration [28].

The normal range of platelet concentration in humans is 150,000 to 450,000 platelets/µL of whole venous blood. PRP produced for clinical use presents predictable benefits when a fourfold increase in platelet concentration is achieved [32]. After platelet activation, growth factor release reaches 70% of the production within 10 min and is maximized, reaching almost 100% within the first hour, while afterward, growth factor secretion continues for up to 8 days. In this way, the PRP activation process should be performed closely to the time of application to maximize therapeutic results [27].

Apart from wound healing properties and the potential for rapid tissue mediated through large concentrations of chemokines and growth factors, PRP also displays an important immunomodulatory role in mesh-augmented surgery [33]. Combined with their direct role in hemostasis and chemotaxis of immune cells, PRP also exerts a series of anti-inflammatory properties in wound healing, such as secretion of hepatocyte growth factor (HGF), reduction in *chemokine receptor CXCR4* expression on inflammatory cells, and reduction of tumor necrosis factor 1a (TNF-1a) and matrix metalloproteinases (MMPs) levels [34].

During the last decades, a large number of studies investigating the applications of PRP as a therapeutic agent in regenerative medicine have emerged. The main fields of PRP clinical use are orthopedics, plastic, and dental and maxillofacial surgery due to its role in the regeneration and healing of cartilage, skin, tendons, and muscles [25]. PRP has been proven to be a safe and effective treatment option for the management of chronic wounds [35]. However, despite the numerous experimental studies on the role of PRP in hernia repair, mainly in combination with mesh implants, a review of their synergic effect is still lacking.

### 1.2. Mesh Coating

Alloplastic implants are a widely used option in hernia surgery and urogynecology [36]. However, mesh-augmented hernia repair is associated with numerous complications, including infection, foreign body reaction, adhesion formation, chronic pain, tearing, and dislocation due to biomechanical mismatch between the implant and the host [37]. More particularly, synthetic implants have been associated with high rates of mesh extrusion, contraction, infection, erosion of adjacent organs, and fistula formation, while biologic grafts are often characterized by impaired tissue incorporation, leading to high mesh-repair failure rates [16].

In order to increase biocompatibility mesh performance, numerous protective materials have been proposed as mesh-coating agents [38]. These mainly include antibacterial biopolymer gels [39], antibiotics [40], antioxidants [40], and blood products, such as peripheral blood mononuclear cells (PBMCs), platelets, and blood plasma [41]. In a scoping review by Baker and Rosenberg, PRP was the second most common coating agent of permanent meshes, providing promising results regarding the improvement of biomechanical properties in hernia repair [42]. More specifically, PRP-augmented meshes present improved post-implantation responses, such as cellular chemotaxis, proliferation, extracellular matrix (ECM) deposition, wound contraction, neovessel formation, and limited immunologic degradation and recurrence rate [43,44]. A review of the effects originating from mesh coating with PRP is presented in Table 2.

## 2. Materials and Methods

### Literature Search Strategy 

Eligible studies were identified by searching the PubMed/Medline, Web of Science, and Scopus electronic databases. A combination if the following terms was used as a search string: platelet-rich plasma (PRP); platelet-rich fibrin (PRF); platelet concentrates; surgical mesh; prostheses; implants; hernia. Potentially relevant articles were initially identified by the title and abstract, and full-text papers were obtained and assessed independently by two researchers (E.A. and D.T.). A manual search of the reference list of each eligible article was also screened to identify further relevant publications. Eligibility criteria included (1) experimental animal (in vivo) studies of repair of ventral, inguinal, diaphragmatic, and hiatal hernias using prosthetic materials coated with platelet products and (2) articles written in English. Exclusion criteria were the following: (1) non-experi-mental studies or in vitro studies, (2) studies using commercially available meshes already coated, and (3) non-English papers. There were no restrictions regarding publication year.

## 3. Experimental Models Based on the Type of Mesh

### 3.1. Synthetic Meshes

Synthetic meshes have been widely utilized for ventral hernia repair in non-contaminated environments [50]. A notable variety of synthetic prosthetics are commercially available, including polypropylene (PP), polytetrafluorethylene (PTFE), dacron, and polyethylene. However, the large number of available synthetic meshes reflects the lack of a single optimal material [51].

Polypropylene mesh (PPM) is the most common and most firmly established type of prosthetic material, with wide use in the fields of abdominal wall and inguinal hernia repair [52]. However, its use and feasibility are affected by host tissue reactions and post-implantation modifications, including cracking, pitting, and flaking. PPM implantation triggers a wide range of tissue responses, including active debridement, incomplete debridement or chronic inflammation, and a foreign-body reaction with increased collagen formation [53]. In addition, oxidative stress (OS) due to decreased tissue perfusion during surgery triggers PPM degradation through inflammatory cell chemotaxis, secretion of reactive oxygen species (ROS), oxidation, and cracking of PPM fibers [54].

An experimental work by Belebecha and colleagues [49] investigated the role of PRP coating after PPM mesh placement on the right side of the abdominal wall in *a New Zealand rabbit model*. After laparotomy and 1 × 1 cm segmental resection, internal and external oblique muscles and the transversalis fascia were removed on both sides. Twelve rabbits were used as a sham group, while the other twelve underwent repair of the left abdominal wall defect only with PPM mesh and repair of the right side with PRP-coated PPM. Euthanasia followed at 30 and 60 days. During laparotomy, macroscopic assessment of adhesion tenacity and extent, as well as the extent of area presenting inflammatory infiltration, revealed no significant difference. Regarding inflammatory response, no difference was noticed among groups at 30 days. However, after 60 days, PRP-coated PPM presented decreased myeloperoxidase (MPO) and N-acetylglucosaminidase (NAG) activities. Finally, for assessing OS, ferric-reducing antioxidant power (FRAP), 3-ethylbenzothiazoline-6-sulfonic acid [2,2′-azino-bis (ABTS), reduced glutathione (GSH), and superoxide anion levels (nitroblue tetrazolium-NBT) were measured. Results revealed a significant increase in antioxidant levels in the PRP-coated group compared to the non-coated group by reducing the loss of endogenous antioxidants and production of reactive oxygen species (ROS). The authors concluded that PRP-covered PPM is a promising adjuvant for abdominal wall healing due to the reduction in oxidative stress and inflammatory response.

Platelet-rich fibrin (PRF) belongs to the second generation of platelet concentrates and constitutes a resorbable fibrin, which provides a controlled release of cytokines, growth factors, and cells [55]. Based on the increasing role of PRF in regenerative procedures, in 2020, El-Husseiny et al. [47] investigated the effect of polypropylene mesh (PPM), glycerolized bovine pericardium (GBP), autologous PRF, and their combination in goats with iatrogenic large mid-ventral abdominal wall defects. Nine animals were included in each group, and euthanasia followed after 4, 8, and 12 weeks. The qualitative and quantitative ultrasonographic evaluation of implant sites performed by the researchers at postoperative days 1, 1, 2, 3, 4, 8, and 12 weeks is important, revealing significant improvement of implant gray scale, reduced subcutaneous edema and skin–implant distance in groups treated with PRF. The latter also presented improved connective tissue deposition and mesh incorporation, increased angiogenesis rate, and reduced inflammatory cell concentration. Tensiometric evaluation, at 8 and 12 weeks postoperatively, including tensile strength (TS), load at failure (LF), and strain percent, were improved in the GBP–PRF treated group compared to the rest of the groups. Results were suggestive of the superiority of GBP over PPM as a prosthetic but also validated the significant role of PRF-augmented compared to non-augmented meshes for hernia repair.

The potential regenerative properties of PRP combined with PPM also aroused the interest of Avila et al. [56], whose experimental study focused on the changes caused by the implantation of PRP-coated PPM regarding the production of collagen I and III and inflammatory infiltrate (ININ). Thirty adult female *New Zealand rabbits* were divided into two groups and underwent subaponeurotic PPM mesh implantation, with or without PRP coating. Euthanasia time points were 7, 30, and 90 days. Histological examination demonstrated no significant difference in the level of inflammatory cells in the initial phase of the experiment (7 days). However, ININ steadily increased after 30 days, achieving a significant difference at 90 days. This indicates the important role of PRP enrichment during the late stages of wound healing. Similarly, PRP coating promoted an increase in concentration of collagen I, collagen III, and total collagen on the 7th postoperative day.

Recently, in 2023, Zedan et al. [57] investigated the role of PRF as an adjuvant factor for hernioplasty performed in a modified sublay fashion with polypropylene mesh in a sheep experimental model. Extensive seroma was noted in animals treated only with mesh, compared to mild seroma noted in animals of the PRP–mesh group. Moreover, deposition and maturation of collagen fibers, granulation tissue formation, and expression of IL-6 and IL-12 were presented earlier in the PRF group, compared to the control group. The authors concluded that the addition of PRF in polypropylene mesh hernioplasty led to reduced inflammation and an important improvement in the healing process.

### 3.2. Biological Implants

Synthetic implants used for hernia repair have been connected with numerous adverse effects, such as infection, erosion, adhesion, migration, and contraction, that limit repair endurance [58,59]. Acellular dermal matrix (ADM), derived from animal or cadaveric tissues, has emerged as a valuable alternative to synthetic meshes for ventral hernia repair [60]. ADMs provide a biomimetic scaffold for further cellular enhancement and proliferation. Their application is of great use under contaminated conditions, but they present significant heterogeneity regarding cellular infiltration, encapsulation, degradation, and host response [17]. Further, biological materials have greater biocompatibility and have been associated with fewer complications [16]. However, biological scaffolds present decreased long-term mechanical strength and higher recurrence rates due to the degradation of the acellular extracellular matrix by the host’s immune system, combined with high costs [61]. More specifically, the persistence of inflammation even after the inflammatory phase of wound healing and the presence of chronic lymphocytic infiltrates may predispose to reduced mechanical strength [34]. In addition, their use in cases of incomplete fascial defects predisposes to hernia recurrence after complete implant incorporation [62].

Early in 2012, Heffner and colleagues [63] delved into the regenerative role of PRP in mesh-augmented surgery as part of their experimental study, which included midline ventral hernia primary repair (Group 1), repair with a bovine collagen implant (CollaTapeTM-CoTa) and PRP (Group 2), or combination of the latter option with bone marrow-derived mesenchymal stromal cells (BM-MSCs) (Group 3). Forty-two *Lewis rats* were used for the needs of the experiment. Focusing on Groups 1 and 2, biomechanical tests revealed a 101% increase in the average tensile strength of Group 2 at 4 weeks and a 38% increase at 8 weeks, compared to Group 1. In addition, Group 2 presented significantly increased modulus of elasticity, modulus of toughness, and energy absorption at both time points. However, the addition of CoTa and PRP (Group 2) did not manage to ameliorate vascularization rates or to produce a significant difference in collagen organization and amount, compared to Group 1 at both euthanasia times. It is also worth mentioning the significantly increased muscle degeneration rate encountered in Group 2 at 4 weeks, compared to Group 1.

Cross-linking of collagen materials is conducted with chemical and physical techniques and aims to increase fibroblast growth and decrease degradation by collagenase enzymes [64]. However, it has been shown that non-crosslinked ADM presents increased cell infiltration and neovascularization, while cross-linking of human cadaveric ADM leads to lower rates of angiogenesis, tissue formation, and host inflammatory response, thus potentially decreasing its biocompatibility, tissue integration, and mechanical strength [65]. Harth et al. reported a series of adverse effects after the use of cross-linked ADMs for abdominal wall reconstruction, including acute mechanical failure, evisceration, and poor integration, necessitating reoperation, probably due to the limitation of cellular and neovessel infiltration [66].

In 2015, Fernandez-Moure and colleagues [67] examined the angiogenetic and proliferative effects of PRP on Strattice, a non-crosslinked porcine ADM, in a ventral hernia rat model. Twenty-eight days after iatrogenic ventral hernia, 42 male *Lewis rats* in total underwent defect repair with non-crosslinked porcine acellular dermal matrix, enhanced with saline or autologous PRP. Euthanasia followed after 2, 4, and 6 weeks. Macroscopic neovessel ingrowth, assessed by the percentage of mesh surface covered with neovessels and tissue, was more profound in animals treated with PRP at all time points, especially at 6 weeks. Furthermore, neovascularization, as determined by CD31 immunohistochemical staining and number of vessels per field of view, confirmed the statistically significant difference in favor of the PRP-treated group at 2, 4, and 6 weeks, with a concurrent increase in vessel size at 6 weeks. Results from histological analysis showed increased thickness of newly formed tissue at 4 and 6 weeks. In addition, defect healing in the PRP was characterized by denser newly formed collagen fibers compared to the saline group, while only PRP groups presented muscle island formation, finally leading to 1.5-fold greater tissue thickness compared to saline groups. The results of this study support the promising role of PRP-coated biological meshes in improving mechanical properties and stability after bridging hernia repair.

The properties of Strattice ADM after PRP coating were explored in 2015 by Van Eps et al. [16], who used a *Lewis rat* model to create an abdominal wall defect simulating a chronic ventral hernia. The authors compared two major groups, one consisting of rats undergoing repair with Strattice mesh alone and one receiving the same mesh coated with autologous PRP. Tissue specimens were harvested at 3 and 6 months postoperatively. At 3 months postoperatively, significantly increased neovascularization of implanted mesh, enhanced tissue ingrowth, and limited chronic immune cell infiltration were found in the mesh–PRP group, compared to the group treated only with mesh. In addition, the molecular investigation revealed significantly increased expression of angiogenic genes (*vEGF* 2.73-fold, *vWF* 2.21-fold) and myofibroblastic genes (*aSMA* 9.68-fold, *FSP-1* 3.61-fold, *Col1a1* 3.32-fold, *Col31a1* 3.29-fold) in the mesh–PRP group, compared to the control group. The mesh–PRP treated rats presented less severe peritoneal at both euthanasia time points, as well as increased mesh thickness improved mesh preservation. Finally, no hernia recurrence was found in the mesh–PRP group at 6 months, compared to 7 recurrence cases among 10 rats of the control group.

Two years later, the same team of Van Eps et al. [68] also explored the potential positive effect of PRP on the incorporation and preservation of mechanical properties of a non-crosslinked porcine ADM (pADM) for ventral hernia repair in a rodent model. Ultrasound shear wave elastography (US-SWE) was used as a non-invasive imaging modality for the assessment of repair outcomes. Twenty-eight rats in total were divided into two groups and, after iatrogenic ventral hernia creation, underwent repair using either Strattice mesh alone or coated with autologous PRP. US-SWE revealed significantly higher Young’s modulus values in the PRP-treated group at both euthanasia time points. In addition, according to qualitative and quantitative histological examination at 3 months, samples of the pADM–PRP group were characterized by reduced inflammation and improved incorporation along the implant/abdominal wall interface. Notably, after 6 months, the PRP-treated group not only had no hernia recurrence but also presented sufficiently preserved mesh integrity, while all animals treated with uncoated mesh presented either hernia recurrence (4/6) or extreme graft thinning (2/6). The abovementioned findings suggest that Strattice coated with PRP offers a promising synergic effect regarding fascial defect healing and mesh integration.

In 2017, Fernandez-Moure et al. [69] investigated the effect of the cross-linking process of a porcine dermis biological mesh on neoangiogenesis induced by PRP application. A chronic ventral hernia model was used after resection of a full-thickness abdominal midline segment of approximately 2 cm × 3 cm without repair and stapled skin closure. At postoperative day 30, 84 *Lewis rats* in total underwent hernia repair with the use of cross-linked (cADM) or non-crosslinked ADMs (ncADM), and they were further classified based on enhancement with PRP or saline (control). Seven rats from each subgroup underwent euthanasia at 2, 4, and 6 weeks, respectively. Peritoneal adhesions were noted mainly between the omentum and the implant and were more severe in groups enhanced with PRP, especially at 6 weeks, when more than 70% of rats in the cADM–PRP and ncADM–PRP groups presented dense bowel or omental adhesions. This effect can be interpreted by the method of PRP enrichment since meshes were soaked in PRP preoperatively and probably could be avoided by anterior application of autologous PRP. Regarding neovascularization, at 2 and 4 weeks, no difference was noted between saline-treated cADM and ncADM groups, while PRP application led to a significantly higher neoangiogenesis rate in the cADM and ncADM groups compared to control saline groups. The pro-angiogenic effect of PRP enrichment was more profound in the ncADM–PRP group, compared to cADM, and approached its peak within the first 2 weeks after application to equalize the saline-treated control groups by the 6th week. According to the authors, the triggering of the neovascularization cascade by PRP in the early stages can be attributed to the limited action duration of growth factors and chemokines secreted by platelets, including TGF, PDGF, and SDF1a.

Hiatal hernia repair with the use of biologically derived implants has been associated with lower recurrence rates when compared to native tissue repair alone, as well as lower rates of mesh-related erosion and mesh infection [70]. However, the long-term efficacy of biologic grafts remains debatable. The effect of the combination of filtered platelet concentrate (fPC) and biologic graft on hiatal hernia repair was first evaluated in 2017 by Altieri and colleagues [45], who divided sixteen *Yorkshire female pigs* into three groups: seven pigs undergoing typical laparoscopic hiatal hernia repair (group HR), eight pigs undergoing laparoscopic hernia repair with biologic graft (group HRM), and nine pigs undergoing laparoscopic hernia repair enhanced with the application of biologic graft and fPC (group fPC). After 8 weeks, euthanasia followed, and the en-block specimen of the distal esophagus with hiatus was harvested. Tissue evaluation included assessment of collagen deposition, vascularization, and inflammation levels at the graft–hiatal interface, while tensile strength (defined as the strength needed to bring hiatus to failure) was measured. Testing was performed using a material testing system combined with a strain extensometer. Yield force (defined as the ultimate strength at which the tissue was permanently deformed) and Young’s modulus were also calculated. The data obtained revealed a trend toward increased collagen formation, and vascularity of the fPC group was demonstrated without significant difference. Interestingly, the fPC group presented a statistically significant increase in mean chronic inflammation levels, as well as in tensile strength, yield force, and Young’s modulus, compared to HR and HRM groups. No difference was observed in sample thicknesses or sample wet mass among groups. By way of conclusion, the authors believe that enhancement of hiatal hernia repair with a combination of autologous fPC and biologic implants is a safe and promising strategy for wound remodeling and healing.

Acellular Cadaveric Dermis (AlloDerm) is a donated human tissue matrix that can be recellularized and revascularized after implantation, providing better incorporation with adjacent tissues and reduced infection, erosion, and migration, compared to synthetic implants. However, it also presents significant rates of failure, eventration, and recurrence, imposing the need for re-intervention [71]. Aiming at overwhelming the high rates of recurrence, Fernandez-Moure et al. [33] investigated the healing properties of PRP, Alloderm, and their combination by elucidating the immune modulation triggered by PRP on the mesh matrix. The experimental animals were male *Lewis rats*, who underwent surgically a full-thickness abdominal midline fascia/muscle/peritoneum defect measuring 2 cm. A total of 42 rats underwent defect repair with the use of Alloderm in an intraperitoneal underlay fashion, which was previously soaked in activated PRP (21 rats) or sterile saline as a placebo (21 rats). Euthanasia followed after 2, 4, or 6 weeks. The macroscopic evaluation revealed significant thinning and degradation in the placebo group, contrary to the PRP–mesh group. The latter also presented extensive vessel ingrowth at all time points, and mainly at 6 weeks, contrary to the placebo group. Preserved mesh thickness and mechanical integrity in the PRP–mesh group could be also attributed to the trend toward a narrower distribution of collagen III fibers found in the PRP group. Similarly, PRP-treated animals presented increased *Col1a1, Col3a1, Tgfb1,* and *Acta1* gene expression at 2, 4, and 6 weeks compared to controls, in a fashion similar to the native wound healing process, presenting a potential biomimetic healing role of PRP-treated Alloderm. Regarding inflammatory response, PRP-treated Alloderm meshes displayed reduced expression of inflammatory proteins TNF-⍺, NOS-2, ARG-1, and MMP-9 and increased Timp1 expression, suggesting a reduced fibrotic response, as well as significantly reduced ratio of CD8+ T cells to total, compared to Alloderm only rats. In conclusion, coating Alloderm with PRP results in a healing phenotype and enhanced mechanical integrity through the modulation of foreign body reaction and lymphocytic infiltration.

The same team of Fernandez-Moure et al. [48], based on positive results of PRP coating in mesh-augmented hernia repair, further delved into the mechanisms by which PRP enhances mesh incorporation and mechanical strength. The in vitro part of the study included an assessment of the response of bone marrow mesenchymal stem cells (BM-MSCs) from *Lewis rats* to PRP addition. Results showed increased levels of stromal-derived factor (SDF)-1, transforming growth factor-beta (TGF-β,) and platelet-derived growth factor (PDGF) in a dose-dependent manner, enhanced proliferation and myofibroblastic differentiation of BM-MSCs, as well as increased microvessel length and proliferation. Regarding the in vivo study, 48 male *Lewis rats* underwent chronic ventral hernia repair with the use of Strattice mesh enhanced with autologous PRP or saline. Euthanasia and mesh evaluation followed after t 2, 4, and 6 weeks. Histopathological evaluation revealed increased cell infiltration and angiogenesis in PRP-treated animals, which is also confirmed by significantly increased expression of collagen type 1 alpha (COL1a), platelet endothelial cell adhesion molecule 1 (PECAM1), and vascular endothelial growth factor (VEGF). In addition, animals treated with PRP coating displayed significantly increased stiffness compared to the animals treated with saline at 4 and 6 weeks, reflecting an improvement in the mechanical properties of the implant.

Coating of acellular dermal matrices with PRP products has been associated with alleviation of the host inflammatory response and reduced CD8+ cell level in the implant area in previous hernia repair experimental models, enhancing mesh incorporation instead of encapsulation and degradation [67]. Based on the gratifying results of biological grafts coated with PRP regarding mesh incorporation and limitation of inflammatory response in hernioplasty, the research group of Araujo-Gutierrez et al. [72] delved further into the investigation of the optimal PRP concentration. A rat model of ventral hernia was used for the assessment of ADM incorporation and inflammatory cell infiltration after mesh-augmented surgery. More specifically, 36 rats were divided into a control group treated with saline and mesh, or a group that received PRP-coated mesh classified into the following groups depending on PRP concentration: 2 million (PRP-LOW), 200 million (PRP-MID), and 2 billion (PRP-HIGH). The mesh implanted was XCM Biologic Tissue Matrix™ (DSM Biomedical), which is a porcine non-crosslinked ADM. Notably, the PRP-HIGH group presented significantly greater tissue deposition at 4 weeks, while the PRP-MID group showed increased mesh thickness at 2 weeks compared to the control and other treatment groups. The cell infiltration grade reflected a trend toward a dose-dependent response to PRP concentration at both time points, with the PRP-HIGH group presenting significantly higher cell infiltration levels compared to the control group at both 2 and 4 weeks and the PRP-LOW group presenting increased cell infiltration only at 4 weeks. Despite a trend toward increased neovascularization cellular infiltration, PRP did not lead to a statistically significant increase in neovascularization at both the 2- and 4-week timepoints noted in the PRP-MID group; PRP coating did not manage to achieve significant neovascularization at the implant site compared to the control group at any time point. Regarding the impact of various doses of PRP on the immune response, PRP-LOW and PRP-MID groups were characterized by significantly lower CD8+ cell concentrates compared to control groups at 2 and 4 weeks. Additionally, all PRP-treated groups presented significantly reduced multinucleated giant cell infiltration at 2 weeks, while at 4 weeks significant reduction was found only in PRP-HIGH and PRP-MID groups. These observations promote significant changes in the inflammatory cascade and may lead to lower fibrosis and adhesion formation rates. The authors conclude that increasing platelet levels of PRP are associated with improved mesh integration and decreased scaffold degradation, as well as enhanced tissue deposition on the implant site.

### 3.3. Composite Implants

To address the complications associated with synthetic polymers as well as the difficulty in finding a single optimal material, composite implants have emerged. Composite, or barrier-coated, mesh is a dual-sided material characterized by the combination of a synthetic parietal side that provides mechanical strength and a visceral surface that permits tissue ingrowth with minimal adhesion formation through the coexistence of more than one material [73]. The main advantage of their application in clinical practice is the safety provided for use in the intraperitoneal space [46].

Based on the gratifying results of a new composite polyester/cotton fabric (Damour) for hernioplasty in ruminants regarding macroscopic success rates, complication, and recurrence rate [74], Abouelsnar et al. [46] studied the potential role of the combination of allogenic PRP and Damour for the healing of abdominal wall defects in a canine model. Twenty-four *mongrel dogs* were equally divided into two groups and underwent a full-thickness abdominal wall defect of 10 × 6 cm, including muscles and peritoneum. In the control group, repair was performed only with Damour mesh, while the PRP group received the same mesh soaked in allogenic PRP. After 2 and 4 months, the PRP group presented significantly increased neovascularization and less severe adhesion, combined with no hernia recurrence, compared to the control group. Significant collagen deposition and neoangiogenesis in the PRP group were also established by histological evaluation, and overexpression of angiogenic and myofibroblastic genes (*COL1α1, COL3α1, VEGF,* and *TGFβ1*) were observed more frequently in both euthanasia time points. The author concludes by highlighting the potential importance of Damour coated with PRP as a cheap hernioprosthetic material that offers reduced recurrence and complication rates in the field of veterinary general surgery.

All experimental studies are summarized in Table 3, Table 4 and Table 5.

## 4. Applications in Clinical Medicine

The first report of PRP application in reconstructive surgery goes back to 1975 [75]. However, even nowadays, there is a scarcity of evidence regarding the role of PRP-coated implants on hernia repair in human subjects. This observation could be attributed to numerous reasons. Firstly, heterogeneity in patient populations and variability in patient characteristics, such as age, comorbidities, and hernia type, complicate the design and interpretation of clinical trials. In addition, standardization of established protocol regarding preparation, dose, and way of mesh infiltration with PRP across different clinical settings presents technical and logistical difficulties [76]. Published data are mainly case reports and do not delve into safety issues on humans. In addition, no standardized protocols have been established regarding PRP’s way of application or dosage.

Trying to address the need for faster cellular binding and improved integration on abdominal wall defects, Popescu and colleagues [77] evaluated prospectively the effect of PRP or PRF on PPM in 32 patients with different types of abdominal wall defects undergoing open repair. The results showed an improvement and acceleration of mesh integration rate up to 65% in both platelet-derived products, providing a cost-effective and feasible way of improving metrics after hernia repair with PPM.

A recent clinical study on patients was conducted by Paranyak et al. [78], who investigated the effect of autologous PRP augmentation on a nonabsorbable self-fixating ProGrip mesh for laparoscopic repair of large hiatal hernias. In the follow-up after 48 months, of the 54 patients undergoing surgery, only two hernia recurrences were observed. In addition, a significant decrease in the mean gastroesophageal reflux disease-health-related quality of life score was noted, reflecting a positive impact of the therapeutic combination. However, the study did not include a control group.

Based on favorable outcomes of animal studies evaluating PRP-coating in hiatal hernia repair, James et al. [79] launched a feasibility cohort study including 12 patients undergoing large paraesophageal hernia repair with the enhancement of PRP. No significant postoperative complications or hernia were noted, while patients also presented sufficient subjective reflux control. PRP application was safe and added only 5 min to the operative time. However, the PRP application method was not standardized and in addition, no control group was included in the study. A summary of clinical studies investigating the role of PRP as a mesh-coating agent is presented in Table 6.

Apart from its promising role in ameliorating mesh integration and accelerating implant site healing, PRP has also been studied as an alternative option for mesh fixation during hernioplasty, aiming at minimizing chronic pain rates. In this orientation, Di Nikola and team [80] used PRF as a mesh fixation tool in 5 patients undergoing open hernioplasty, concluding that it could provide decreased postoperative pain and minimize recurrence and other complication rates.

## 5. Limitations

The present study constitutes a review of studies investigating the role, benefits, and limitations of mesh coating with PRP for abdominal wall and hiatal hernia repair. The great majority of studies present the same drawbacks, such as poor discussion of long-term outcomes and no standardized PRP concentration and surgical technique. The great heterogeneity of the existing research does not permit the systematic evaluation or quantitative analysis of the data acquired.

Despite the extended preclinical research on the role of mesh coating with platelet-derived products, one should underline the numerous limitations arising from the translation of acquired knowledge to clinical practice. First of all, despite the anatomical similarity of most experimental models to humans, there are still significant differences in hernia pathophysiology, abdominal wall structure, and wound healing that can impact the outcomes of hernia research. In addition, the absence of comorbidities in experimental models included in this review, such as diabetes, obesity, and other chronic conditions that interfere with wound healing, limits the generalizability of the findings to the patient population. Finally, the controlled environment in which animal studies are conducted does not replicate the variability and complexity of clinical settings, in which factors such as patient lifestyle, environmental influences, and genetic diversity also affect postoperative outcomes. In conclusion, results emerging from this review should be cautiously interpreted and validated through clinical trials.

## 6. Conclusions

Complex abdominal wall and hiatal hernia repair constitute a challenging problem for general surgeons. The literature includes numerous studies investigating the effect of mesh enhancement with PRP, mostly concentrating on ventral hernia repair. Coating of meshes with autologous PRP is a simple, low-cost, and safe procedure with positive effects on implantation site angiogenesis, fibroblast proliferation, collagen formation, and deposition. In addition, enhancement of mechanical strength, expressed by increased tensile strength and yield force, has been observed after the use of meshes perpetuated by the PRP components. The addition of PRP to prosthetic matrices leads to improved tissue response, decreased incidence of incisional hernias and hernia recurrence, and increased mechanical strength. This feature is of utmost importance for feature use in patients in whom poor incorporation is anticipated, and early-enhanced neovascularization is desired, such as patients with microangiopathy. In conclusion, PRP-coated mesh augmented hernia repair may lead to more rapid and robust mesh integration and decreased recurrence rate by accelerating the wound-healing process and promoting healthier tissue regeneration than that produced through traditional defect repair. However, further studies are required to ascertain the clinical efficacy and safety of PRP-coated meshes.

## Figures and Tables

**Table 1:** Main growth factors and cytokines deriving from PRP and their functions.

Growth Factors and Cytokines	Functions
platelet-derived growth factor (PDGF) [29]	Proliferation of mesenchymal cells and osteoblasts Regulation of granulocytes, monocytes, and fibroblasts migration and mitogenesis Control of extracellular matrix Regulation of collagenase secretion and collagen synthesis Contribution to bone formation
epidermal growth factor (EGF) [29]	Enhancement of cellular differentiation Proliferation and differentiation of mesenchymal cells
transforming growth factor -β (TGF -β) [30]	Control of cellular mitosis and differentiation Proliferation and differentiation of mesenchymal cells via paracrine action Regulation of collagenase secretion and collagen synthesis Stimulation of endothelial chemotaxis and angiogenesis Inhibition of macrophage and lymphocyte proliferation
insulin growth factor 1 (IGF-1) [30]	Chemotaxis of fibroblasts Stimulation of protein synthesis Proliferation and differentiation of osteoblasts
vascular endothelial growth factor (VEGF) [31]	Increase in angiogenesis and vessel permeability Stimulation of endothelial cell mitogenesis
keratinocyte growth factor (KGF) [30]	Control of epithelial migration and proliferation
platelet factor 4 (PF-4) [30]	Regulation of leucocytes chemotaxis and activation
connective tissue growth factor (CTGF) [30]	Promotion of neoangiogenesis, cartilage formation, fibrosis, and platelet adhesion
tumor necrosis factor (TNF) [30]	Regulation of monocyte migration, fibroblast proliferation, and macrophage activation Promotion of angiogenesis
angiopoietin (Ang-1) [31]	Promotion of angiogenesis Migration and proliferation of endothelial cells. Support of blood vessel development
stromal cell-derived factor -1α (SDF-1α) [31]	Control of CD34+ cells chemotaxis, proliferation and differentiation Promotion of angiogenesis Chemotaxis of mesenchymal stem cells and leucocytes
hepatocyte growth factor (HGF) [30]	Control of cell growth and motility in epithelial/endothelial cells Promotion of epithelial repair and neovascularization
(a-b)- fibroblast growth factor (FGF) [31]	Growth and differentiation of chondrocytes and osteoblasts Promotion of mesenchymal cells, chondrocytes, and osteoblasts mitogenesis

**Table 2:** PRP-mediated activities in synergic mesh–PRP hernia repair.

PRP-Mediated Activities in Synergic Mesh–PRP Hernia Repair
Increase in chronic inflammation [45]
Increase in collagen formation and deposition [45,46]
Increase in new tissue mechanical strength [45]
Εnhancement of angiogenesis [47]
Myofibroblast recruitment and tissue ingrowth [16]
Reduction of CD8+ cell concentrates and multinucleated giant cell infiltration [33]
Improvement of stromal cell migration/proliferation and deposition of the provisional matrix required for sufficient wound healing [45]
Reduction of adhesion formation through fibrinolysis of adhesions and reduced mature transformation [46]
Regulation of chemotaxis of immune cells [16]
Reduction of inflammatory cytokine production [48]
Reduction of loss of endogenous antioxidants [49]
Reduction of matrix metalloproteinase expression [48]

**Table 3:** Basic study characteristics.

Study	Country	Groups—Number of Animals	Animal Type	Age	Weight	Sex	Euthanasia
Heffner et al., 2012 [63]	USA	—42 animals classified into 3 groups: Group 1: primary repair only; Group 2: primary repair with implant and PRP; Group 3: primary repair with implant, PRP, and BM-MSCs	*Lewis rats*	Adult	250–300 gr	M	4 and 8 weeks
Van Eps et al., 2015 [16]	USA	—32 animals classified into 2 groups: mesh only (control) or mesh–PRP —8 animals for blood sampling and PRP production	*Lewis rats*	Adult	Non-stated	M	3 and 6 months
Fernandez-Moure et al., 2015 [67]	USA	—42 animals classified into 2 groups: PRP–mesh group or saline–mesh groups —10 animals for blood sampling and PRP production	*Lewis rats*	Non-stated	300–315 g	M	2, 4, and 6 weeks
Avila et al., 2016 [56]	Brazil	—30 animals classified into 2 groups: mesh-only or PRP-coated mesh	*White New* *Zealand rabbits*	Adult	Non-stated	F	7, 30, and 90 days
Fernandez-Moure et al., 2017 [69]	USA	—84 animals classified into 2 groups: Group 1: Permacol™ cADM and Group 2: Strattice™-ncADM. Groups were afterward divided into PRP or saline-coated (7 animals in each subgroup) —10 animals for blood sampling and PRP production	*Wistar rats*	Non-stated	300–315 gr	M	2, 4, and 6 weeks
Abouelnasr et al., 2017 [46]	Egypt, Japan	—24 animals classified into 2 groups: treated with mesh alone (control group) or mesh and allogenic PRP (PRP group)	*Mongrel dogs*	1.5–2 years	20–30 kg	Non-stated	2 and 4 months
Altieri et al., 2017 [45]	USA	—16 animals classified into 3 groups: hiatus repair (HR) (n = 7), HR with biologic graft (HRM) (n = 8); HR with biologic graft and fPC (fPC; n = 9)	*Yorkshire pigs*	Non-stated	30–40 kg	F	8 weeks
Belebecha et al., 2019 [49]	Brazil	—24 animals classified into 2 groups: sham group and study group, in which mesh was implanted on both sides of the abdominal wall, with only the right side being coated with PRP	*New Zealand rabbits*	6 months	2.91 kg	F	30 and 60 days
Van Eps et al., 2019 [68]	USA	—28 animals classified into 2 groups: pADM alone (pADM group) or pADM coated with PRP (pADM–PRP group)	*Lewis rats*	Non-stated	250–300 g	M	3 and 6 months
El-Husseiny et al., 2020 [47]	Egypt	—36 animals classified into 4 groups (9 animals/group): GBP, PPM, GBP–PRF, and PPM–PRF	*Goats*	1–3 years	20–30 kg	Non-stated	4, 8, and 12 weeks
Fernandez-Moure et al., 2021 [33]	USA	—42 animals classified into 2 groups: PRP–mesh or saline–mesh (placebo)	*Lewis rats*	Non-stated	300–315 g	M	2, 4, and 6 weeks
Fernandez-Moure et al., 2021 [48]	USA	—48 animals classified into 2 groups: PRP–mesh or saline–mesh group —10 animals for blood sampling and PRP production	*Lewis rats*	Non-stated	300–315 g	M	2, 4, and 6 weeks
Araujo-Gutierrez et al., 2021 [72]	USA	—36 animals classified into 4 groups: control (mesh alone, or saline) or experimental (mesh + PRP), including three different PRP concentrations: 2 million (PRP-LOW); 200 million (PRP-MID); 2 billion (PRP-HIGH)	*Lewis rats*	Non-stated	300–315 gr	M	2 and 4 weeks
Zedan et al., 2023 [57]	Iraq	—24 animals classified into 2 groups: PPM only (control group) and PPM reinforced with PRF (PRF group)	*Sheep*	12 ± 0.2 months	41 ± 0.4 kg	M	7, 15, 30, 45 days

PRP: platelet-rich plasma; PPM: polypropylene mesh; GBP: glycerolized bovine pericardium; PRF: platelet-rich fibrin; BM-MSCs: bone marrow-derived mesenchymal stromal cells; M: male; F: female; pADM: porcine acellular dermal matrix; fPC: filtered platelet concentrate; gr: grams; kg: kilograms; cADM: crosslinked ADM; ncADM: non-crosslinked ADM

**Table 4:** Characteristics of defects and mesh-related details of included studies.

Author	Type of Hernia	Defect Characteristics	Mesh	Type of Mesh	Mesh Dimensions	Fixation Method	Mesh Location
Heffner et al., 2012 [63]	Ventral hernia	6 cm midline full-thickness fascial defect	CollaTapeTM (CoTa) (Zimmer Dental, Mississauga, ON, Canada)	type I bovine collagen	1 × 6 cm	5–0 Vicryl sutures	Onlay
Van Eps et al., 2015 [16]	Ventral hernia	2 cm full-thickness incision of the abdominal wall at the linea alba	Strattice (Life Cell Corporation, Branchburg, NJ, USA)	ncADM	Non-stated (at least a 1.5 cm overlap from the edge)	Eight interrupted 3–0 Prolene sutures	Intraperitoneal underlay
Fernandez-Moure et al., 2015 [67]	Ventral hernia	Full-thickness (fascia/muscle/peritoneum) abdominal midline defect measuring 2 cm in length	Strattice (Life Cell Corporation, Branchburg, NJ, USA)	porcine ncADM	2.5 × 1.5 cm	Eight transfascial 5–0 Prolene sutures	Intraperitoneal underlay
Avila et al., 2016 [56]	Ventral hernia	Dimensions not mentioned	Brand non-stated	PPM	1 × 1 cm	No fixation	Underlay (subaponeurotic)
Fernandez-Moure et al., 2017 [69]	Ventral hernia	Full-thickness abdominal midline defect measuring 2 cm × 3 cm	cADM: Permacol (Medtronic, Minneapolis, MN, USA), ncADM: Strattice (Life Cell, Bridgewater, NJ, USA)	cADM and ncADM	Non-stated	Transabdominal polypropylene sutures through fascia and abdominal musculature	Underlay
Abouelnasr et al., 2017 [46]	Ventral hernia	Full-thickness abdominal wall defect measuring 10 × 6 cm and including muscles and peritoneum	Damour (Further information are not provided)	Polyester/cotton fabric	Covering the edges with 5–8 mm underlay	Interrupted 1–0 Polypropylene monofilament sutures	Underlay
Altieri et al., 2017 [45]	Hiatal hernia	1.5 cm hiatal opening	Cook Biodesign 4-layer hiatal hernia graft (Cook, Bloomington, IN, USA)	ADM	7 × 10 cm	Two 2–0 silk sutures	Hiatus
Belebecha et al., 2019 [49]	Ventral hernia	1 × 1-cm partial thickness (internal and external oblique muscles and transversalis fascia) paramedian defect	Advantage (Boston Scientific, Marlborough, MA, USA)	PPM	1.5 × 2 cm	Four interrupted 4–0 polyglycolic acid sutures	Between the hypodermis and peritoneum
Van Eps et al., 2019 [68]	Ventral hernia	A full-thickness 2 cm defect, including the peritoneum	Strattice (LifeCell Corporation, Branchburg, NJ, USA)	porcine ncADM	2.5 x 3 cm	Eight interrupted, 5–0 Prolene sutures	Intraperitoneal onlay
El-Husseiny et al., 2020 [47]	Ventral hernia	Full-thickness abdominal wall defect, including the peritoneum measuring 6 × 10 cm	Heine Mesh, (30 cm × 30 cm, HEINE MEDIZIN GmbH., Dusseldorf, Germany)	PPM	Non-stated	Interrupted chromic cat gut size 0 sutures	Intraperitoneal underlay
Fernandez-Moure et al., 2021 [33]	Ventral hernia	Full-thickness abdominal midline fascia/muscle/peritoneum defect measuring 2 cm in length	Alloderm (Lifecell Corporation, Branchburg, NJ, USA)	human ncADM	2.5 × 1.5 cm	Six transfascial 5–0 Prolene sutures	Intraperitoneal underlay
Fernandez-Moure et al., 2021 [48]	Ventral hernia	Full-thickness abdominal midline fascia/muscle/peritoneum defect measuring 2 cm in length	Strattice (LifeCell Corporation, NJ, USA)	pADM	3 × 3 cm	Interrupted transfascial 5–0 Prolene sutures	Intraperitoneal underlay
Araujo-Gutierrez et al., 2021 [72]	Ventral hernia	Full-thickness abdominal midline incision through fascia, muscle, and/or peritoneum measuring 2 cm	XCM Biologic Tissue Matrix (DSM Biomedical, Exton, PA, USA)	porcine ncADM	2.5 × 1.5 cm	Eight transfascial 5–0 prolene (Ethicon) sutures	Intraperitoneal underlay
Zedan et al., 2023 [57]	Ventral hernia	12 cm vertical incision through the skin and subcutaneous tissue without peritoneum opening	Monoprolen (Betatech Medical, Instabul, Turkey)	PPM	30 × 30 cm	Non-stated	Modified sublay

ADM: Acellular Dermal Matrix; PPM: polypropylene mesh; pADM: porcine acellular dermal matrix; cADM: crosslinked ADM; ncADM: non-crosslinked ADM; cm: centimeters.

**Table 5:** Main results and conclusions of included studies.

Author	*Animal Type*	Main Results	Conclusions
Heffner et al., 2012 [63]	*Lewis rats*	—Group 2 presented increased tensile strength at 4 and 8 weeks compared to Group 1΄ —Group 2 presented significantly increased modulus of elasticity, modulus of toughness, and energy absorption at both time points compared to Group 1 —At 4 weeks, Group 2 presented significantly increased muscle degeneration rates compared to Group 1 —No significant difference in neovascularization rates and in collagen organization and amount between the two groups	PRP-coated collagen matrix led to improved rates of biomechanical tests without, however, improving neovascularization or quality and quantity of collagen deposition
Van Eps et al., 2015 [16]	*Lewis rats*	—No significant difference in seroma formation in each group —At 3 months: Mesh–PRP groups presented significantly increased neovascularization, upregulation of both angiogenic and myofibroblastic genes, increased tissue deposition, and reduced chronic immune cell infiltration compared to mesh group —Peritoneal adhesions were less severe at both 3 and 6 months in the mesh–PRP groups —Mesh–PRP group had no hernia recurrence rate at 6 months, compared to mesh group (7/10) and presented significantly improved mesh preservation	PRP coating induced angiogenesis, myofibroblast recruitment, and newly formed tissue ingrowth leading to improved ADM preservation, reduced rate of severe peritoneal adhesions, and diminished hernia recurrence rate
Fernandez-Moure et al., 2015 [67]	*Lewis rats*	—PRP–mesh animals presented increased neovascularization, both macroscopically and on immunohistochemical analysis, and improved mesh incorporation at all time points compared to saline–mesh group —PRP–mesh group displayed increased thickness of tissue deposition at 4 and 6 weeks	—PRP coating led to increased neovascularization and improved incorporation —Enhanced neovascularization triggered by PRP was correlated with faster and greater newly formed tissue deposition
Avila et al., 2016 [56]	*White New* *Zealand rabbits*	—At 90 days, significant increase in the number of inflammatory cells in PRP group, compared to mesh-only group —At 7 days, PRP group presented increased production of collagen I, III, and total compared to mesh-only group	PRP-coating resulted in greater inflammatory cell infiltration at the implant site and increased collagen concentration
Fernandez-Moure et al., 2017 [69]	*Wistar rats*	—Seromas presented in 12 animals of cADM–PRP group, in 13 animals of ncADM–PRP group, in 7 animals of cADM, and 6 animals of ncADM —PRP coating led to increased neoangiogenesis in both cADM and ncADM groups at 2 and 4 weeks, with significantly increased rates in ncADMs compared to cADMs —Adhesions were increased and more severe in all PRP-treated groups	—PRP enhances native tissue response and early neovascularization of implant sites —ncADM is more amenable than cADM to PRP-triggered neovascularization
Abouelnasr et al., 2017 [46]	*Mongrel dogs*	—Seroma formation was more common in control group compared to PRP group, with no significant difference —Wound dehiscence and infection were observed only in two dogs from the control group —PRP-treated dogs presented significantly increased neovascularization and less severe adhesions compared to control group —PRP-treated dogs presented no hernia recurrence —Histological and molecular tests confirmed increased collagen deposition, neoangiogenesis, and overexpression of angiogenic and myofibroplastic genes in the PRP group at both time points	PRP-coated Damour presented increased neoangiogenesis and tissue deposition, improved graft incorporation, reduced peritoneal adhesions, and diminished hernia recurrence rate
Altieri et al., 2017 [45]	*Yorkshire pigs*	—No difference in sample thicknesses, sample wet mass, and vascular deposition among groups —fPC group presented significantly increased mean chronic inflammation and increased tensile strength, yield force, and Young’s modulus, compared to HR and HRM groups —A trend toward increased collagen deposition was demonstrated in fPC group without significant difference	fPC mesh enhancement presented significantly increased mean chronic inflammation and improved biomechanical metrics, as well as a trend toward increased collagen deposition and vascularity compared to primary hiatus repair and repair with biologic implant only
Belebecha et al., 2019 [49]	*New Zealand rabbits*	—No significant difference between sham and study groups regarding adhesions at 30 and 60 days —MPO activity was significantly reduced at 60 days on the PRP-coated side. At 60 days, PRP-coated group presented a significant reduction in NAG activity —At 60 days, the PRP presented a reduction in GSH levels and a significant increase in superoxide anion production compared to the uncoated side —PRP coating led to a significant increase in antioxidant levels compared to the uncoated side	Coating of PPM with PRP led to a reduction in OS levels and inflammatory responses without affecting adhesion formation
Van Eps et al., 2019 [68]	*Lewis rats*	—pADM–PRP group presented significantly higher Young’s modulus values in ultrasound shear wave elastography at both euthanasia time points compared to pADM group —pADM–PRP group displayed reduced inflammation and improved incorporation along the implant/abdominal wall interface at 3 months, while after 6 months, this group had no hernia recurrence and presented preserved mesh integrity, while all animals treated with uncoated mesh presented either hernia recurrence (4/6) or extreme graft thinning (2/6)	PRP coating leads to reduced inflammation, improves mesh integration, and diminishes hernia recurrence rate when it is used for pADM coating
El-Husseiny et al., 2020 [47]	*Goats*	—PRF-treated groups presented significant improvement in ultrasonographic findings and improved connective tissue deposition, mesh incorporation, increased angiogenesis, and reduced inflammatory cell concentration —Improved tensiometric tests were noted in GBP–PRF treated group compared to other groups	Implant enhancement with PRF was superior compared to the use of implant only, through increased neovascularization and tissue deposition, improved implant incorporation, reduced inflammatory response and complications
Fernandez-Moure et al., 2021 [33]	*Lewis rats*	—PRP–mesh group did not exhibit significant degradation or thinning and presented more evident vascular ingrowth compared to placebo group —Increased PRP results in decreased inflammatory cytokine production, decreased matrix metalloproteinase expression, and decreased CD8+ T cell infiltration —Increased stiffness of implanted mesh was noted in PRP–mesh group	Alloderm coating with PRP temporally modulates the innate and cytotoxic inflammatory reactions to the mesh, resulting in decreased inflammatory cytokine production early postoperatively, reduced matrix metalloproteinase expression, and decreased CD8+ T cell infiltration in the implant site, thus promoting a healing phenotype toward reduced mesh thinning and increased material stiffness
Fernandez-Moure et al., 2021 [48]	*Lewis rats*	—Specimen from PRP-treated animals presented increased cell infiltration and angiogenesis, increased expression of COL1a, PECAM1, and VEGF —PRP-coated meshes displayed increased stiffness compared to the animals treated with saline at 4 and 6 weeks	PRP coating enhances cell recruitment, proliferation, and angiogenesis, resulting in improved tissue regeneration, mesh incorporation, and mechanical strength
Araujo-Gutierrez et al., 2021 [72]	*Lewis rats*	—PRP-HIGH group had significantly greater tissue deposition at 4 weeks —PRP-MID showed increasing mesh thickness at 2 weeks —Cell infiltration was significantly higher with PRP-HIGH at both 2 and 4 weeks, while PRP-LOW showed increased cell infiltration only at 4 weeks —No statistically significant differences in neovascularization were found —CD8+ cell infiltrate was significantly decreased at 2 and 4 weeks in PRP-LOW and PRP-MID treated groups —All PRP-treated groups presented significantly decreased MNGC infiltration at 2 weeks. Only PRP-HIGH and PRP-MID groups had a significant reduction in MNGC at 4 weeks	Increasing platelet concentrations resulted in improved graft incorporation and tissue deposition, as well as reduced mesh scaffold degradation, due to blunted foreign body response and reduced inflammation
Zedan et al., 2023 [57]	*Sheep*	—Developing seroma in control group, compared to mild seroma in PRF–mesh group —Deposition and maturation of collagen fibers, granulation tissue formation, mononuclear inflammatory cells infiltration, hyperplasia of fibrocytes, collagen deposition, and edema presented earlier in the mesh–PRF group, compared to control group —Expression of IL-6 and IL-12 began earlier in the mesh–PRF group than in the control group	PPM enhancement with PRF led to reduced inflammation and improved histopathological and immunohistochemistry findings

PRP: platelet-rich plasma; PPM: polypropylene mesh; GBP: glycerolized bovine pericardium; Il-6: interleukin-6, IL-12: Interleukin-12; PRF: platelet-rich fibrin; MNGC: multinucleated giant cell; OS: oxidative stress; pADM: porcine acellular dermal matrix; fPC: filtered platelet concentrate; COL1a: collagen type 1 alpha; PECAM1: platelet endothelial cell adhesion molecule 1; VEGF: vascular endothelial growth factor; MPO: myeloperoxidase; NAG: N-acetylglucosaminidase; GSH: glutathione; ADM: Acellular Dermal Matrix; cADM: crosslinked ADM; ncADM: non-crosslinked ADM.

**Table 6:** Clinical studies investigating the role of mesh coating with PRP.

Author	Type of Study	Number of Patients	Type of Hernia	PRP Use	Main Results
Popescu et al., 2021 [77]	Prospective comparative study	32 patients, classified into 3 groups: standard procedure; mesh+PRF; mesh+PRP	Different types of abdominal wall defects	Coating	Addition of plasma-derived products led to improved and faster mesh integration, compared to standard procedure
Paranyak et al., 2021 [78]	Prospective cohort study	54 (no control group)	Hiatal	Coating	Infiltration with PRP led to positive results, including low recurrence rate and lower mean gastroesophageal reflux disease-health-related quality of life score
James et al., 2023 [79]	Prospective cohort study	12 (no control group)	Paraesophageal	Coating	PRP-treated patients presented good subjective reflux control, with no significant postoperative complications or hernia recurrence

PRF: platelet-rich fibrin.
